# Breast Contrast Enhanced MR Imaging: Semi-Automatic Detection of Vascular Map and Predominant Feeding Vessel

**DOI:** 10.1371/journal.pone.0161691

**Published:** 2016-08-29

**Authors:** Antonella Petrillo, Roberta Fusco, Salvatore Filice, Vincenza Granata, Orlando Catalano, Paolo Vallone, Maurizio Di Bonito, Massimiliano D’Aiuto, Massimo Rinaldo, Immacolata Capasso, Mario Sansone

**Affiliations:** 1 Division of Radiology, Department of Diagnostic Imaging, radiant and metabolic Therapy, “Istituto Nazionale Tumori Fondazione Giovanni Pascale–IRCCS”, Via Mariano Semmola, Naples, Italy; 2 Division of Diagnostic Pathology, Department of Diagnostic and Laboratory Pathology “Istituto Nazionale Tumori Fondazione Giovanni Pascale–IRCCS”, Via Mariano Semmola, Naples, Italy; 3 Divison of Senology Surgery, Department of Senology “Istituto Nazionale Tumori Fondazione Giovanni Pascale–IRCCS”, Via Mariano Semmola, Naples, Italy; 4 Department of Electrical Engineering and Information Technologies, University “Federico II” of Naples, Via Claudio, Naples, Italy; Generalitat Valenciana, SPAIN

## Abstract

**Purpose:**

To obtain breast vascular map and to assess correlation between predominant feeding vessel and tumor location with a semi-automatic method compared to conventional radiologic reading.

**Methods:**

148 malignant and 75 benign breast lesions were included. All patients underwent bilateral MR imaging. Written informed consent was obtained from the patients before MRI. The local ethics committee granted approval for this study. Semi-automatic breast vascular map and predominant vessel detection was performed on MRI, for each patient. Semi-automatic detection (depending on grey levels threshold manually chosen by radiologist) was compared with results of two expert radiologists; inter-observer variability and reliability of semi-automatic approach were assessed.

**Results:**

Anatomic analysis of breast lesions revealed that 20% of patients had masses in internal half, 50% in external half and the 30% in subareolar/central area. As regards the 44 tumors in internal half, based on radiologic consensus, 40 demonstrated a predominant feeding vessel (61% were supplied by internal thoracic vessels, 14% by lateral thoracic vessels, 16% by both thoracic vessels and 9% had no predominant feeding vessel—*p*<0.01), based on semi-automatic detection, 38 tumors demonstrated a predominant feeding vessel (66% were supplied by internal thoracic vessels, 11% by lateral thoracic vessels, 9% by both thoracic vessels and 14% had no predominant feeding vessel—*p*<0.01). As regards the 111 tumors in external half, based on radiologic consensus, 91 demonstrated a predominant feeding vessel (25% were supplied by internal thoracic vessels, 39% by lateral thoracic vessels, 18% by both thoracic vessels and 18% had no predominant feeding vessel—*p*<0.01), based on semi-automatic detection, 94 demonstrated a predominant feeding vessel (27% were supplied by internal thoracic vessels, 45% by lateral thoracic vessels, 4% by both thoracic vessels and 24% had no predominant feeding vessel—*p*<0.01). An excellent agreement between two radiologic assessments (k = 0.81) and between radiologic consensus and semi-automatic assessment (k = 0.80) was found to identify origin of predominant feeding vessel. An excellent reliability for semi-automatic assessment (Cronbach's alpha = 0.96) was reported.

**Conclusions:**

Predominant feeding vessel location was correlated with breast lesion location: internal thoracic artery supplied the highest proportion of breasts with tumor in internal half and lateral thoracic artery supplied the highest proportion of breasts with lateral tumor.

## Introduction

Magnetic resonance imaging (MRI) has become an important imaging modality in the breast cancer diagnostic work-up. MR is the most accurate imaging method for non-invasive breast cancer detection, having nearly 100% sensitivity [1]. The specificity, however, is only moderate reaching an overall specificity of 72% in 44 studies as reported by Peters et al. [1] and varies widely across studies in relation with cancer prevalence and the criteria used to differentiate malignant and benign lesions [1].

Breast Contrast Enhanced Magnetic Resonance Imaging (CE-MRI) provides the study of breast vascular map by means of the enhancement of an injected paramagnetic contrast agent. Maximum intensity projections (MIPs) obtained from post-processing of subtraction images can reveal not only the presence of enhancing lesions but also the angiographic vessel vascular map of the whole bilateral breast.

The diagnostic value of breast vascular maps has recently been explored [2–3] and an increased vascularity adjacent to lesion and in the ipsilateral breast as a whole has been found [4–9]. The presence of vessels adjacent to one or more lesions has been investigated in several papers [7, 10]. In 2002, a study by Carriero et al. [5] reported 89% of sensitivity and 83% of specificity for perilesional or intralesional vessels. Kul et al. [7] showed that both ipsilateral increased vascularity (sensitivity and specificity of 62% and 79%, respectively) and the adjacent vessel sign (sensitivity and specificity of 74% and 89%, respectively) are associated with breast cancer in a significant percentage of patients. In 2005, Malich et al. [10] defined the adjacent vessel as “a prominent vessel leading to the lesion, seen in subtraction images” and reported it as “a minor sign of malignancy” present in 63% of 268 malignant lesions and in 28% of 60 benign lesions. Fischer et al. [11] obtained a sensitivity of 83% and a specificity of 87% on 122 patients (71 malignant lesion and 51 benign lesion). Dietzel et al. [12] examined the diagnostic value of the adjacent vessel on a large population (1.084 cases) reporting a sensitivity of the adjacent vessel sign of 47%, a specificity of 88%, a positive likelihood ratio of 3.8, a negative likelihood ratio of 0.6 and diagnostic odds ratio of 6.3. Finally, Grubstein et al. [13] characterized the alterations in blood supply by location of the tumor (medial versus lateral) within the breast using MRI. The study involved 105 patients with a cancer prevalence of 49%. A dominant vessel was noted in 47 of 51 cases (92%). A predominant medial vascular supply, connected to the internal mammary vessels, was seen in 87% of medial tumors and 48% of lateral tumors.

In most studies present in literature, the critical issues were the subjectivity of visual methods for vessel analysis, the quantification suffered from intra- and inter-observer variability and the approach could be time consuming considering the need to measure the size of each vessel and then to identify the predominant feeding vessel. In this scenario, it is desirable the development of a software dedicated to the semi-automatic detection of vessels within the breast and of the predominant feeding vessel. Moreover, it is desirable to assess the correlation between breast cancer site and predominant feeding vessel location.

In this study, we propose a semi-automatic method to obtain the breast vascular map and the predominant feeding vessel and to correlate the latter with breast lesion location. We have compared the findings of the semi-automatic approach with the results of two readers expert radiologists and we have further evaluated the inter-observer variability and a measure of semi-automatic approach reliability.

## Material and Methods

### Study population

Retrospectively, a single-institution database was reviewed for patients diagnosed with breast cancer and who were surgically treated for breast cancer between 2011 and 2014. The records of 3499 consecutive patients were analyzed. Of these, 298 patients underwent breast contrast-enhanced MRI, between 7^th^ and 14^th^ day of menstrual cycle for women in premenopausal phase, with a dedicated equipment because of abnormal mammographic or ultrasound or clinical findings. Among these, patients with unilateral and unifocal histo-pathologically-confirmed lesions were included in this study. Patients with bilateral or multifocal breast cancer or with a history of radiation therapy or breast biopsy within 6 months or with no histologic confirmation of the lesion, who had undergone unilateral mastectomy for a previous diagnosis of breast cancer, or who had undergone unilateral imaging were excluded by consensus of two expert radiologists. An overall of 223 patients (age range, 20–73 years; mean, 45 years), with a defined breast lesion, visible vessels on contrast enhanced MR acquisitions and no evident motion artifacts on subtraction images were included. 148 had a malignant tissue diagnosis and 75 had a benign tissue diagnosis. All patients underwent bilateral imaging. Written informed consent was obtained from each patient before MRI. No personal medical information about an identifiable living individual was presented in this study. Approval for this study was granted by the ethics committee of National Cancer Institute of Naples “Pascale Foundation”.

### Magnetic Resonance Imaging Protocol

Dynamic Contrast Enhanced Magnetic Resonance Imaging was performed with a 1.5T breast dedicated system (Aurora; Aurora Imaging Technology, North Andover, MA), enclosing an integrated in-table coil [14]. A pre-contrast three-dimensional (3D) non-spoiled SPIRAL-RODEO fat-sat sequence (TR 29 ms, TE 4.8 ms, flip angle 45°, matrix 512x512, thickness 1.13 mm, gap 0 mm) and four dynamic post-contrast 3D spoiled SPIRAL-RODEO fat-sat acquisitions (TR 29 ms, TE 4.8 ms, flip angle 45°, matrix 512x512, thickness 1.13 mm, gap 0 mm) were obtained with temporal resolution of 90 seconds (160 slice to encompass the entire volume of interest). Gadobenate dimeglumine (Multihance, Gd-BOPTA Bracco; AtlantaPharma, Costance, Germany) was administered intravenously, as a bolus injection, at a dose of 0.2 mL⁄kg body weight, followed by flushing with 20 mL of saline via an Optistar Elite (Covidien Imaging Solution, Hazelwood, MO) automated contrast delivery system.

### Histopathologic Analysis and Reference Standard

Breast pathologists with experience of at least 10 years performed histopathological verification. Pathology of surgical specimens served as reference standard for imaging findings. Benign breast lesions were divided into the following subcategories: fibroadenoma, phyllodes tumor, papilloma and fibrocystic changes. Malignant lesions were histopathologically classified according to the World Health Organization (WHO) classification of breast carcinoma. The size of the tumor was measured across the largest cross sectional dimension. The intensity, extent, and subcellular distribution of estrogen receptor (ER), progesterone receptor (PR) and human epidermal growth factor receptor 2 (HER2) were evaluated as previously described by Crispo et al [15].

### Image Analysis

#### Radiologic Evaluation

Two dedicated radiologists with 20 years of experience (A.P.) and 15 years of experience (S.F.) interpreted breast MRI scans. The radiologists provided subjective evaluation of images both individually and in consensus; in particular, they proceeded by means of visual inspection of the vertical Maximum Intensity Projection (MIP) of the subtraction between the first post-contrast (1.5 min after IV injection) and the pre-contrast acquisition.

The radiologists were uninformed about the pathological findings. The evaluation focused on two main aspects:

1. Presence of a mass and location of lesion (internal, external half and subareolar/central area).
2. Presence of a dominant feeding vessel and origin of the vessel (internal, lateral or internal/lateral thoracic artery).

To individuate the dominant feeding vessel each radiologist proceeded as follows: each reader evaluated the MIPs of subtraction images from several different angles of projection in order to become acquainted with the vascular map of the patient; then he/she subjectively individuated the dominant vessels. The qualitative criteria used for establishing the presence of dominant vessels were both a “sufficient” (in the opinion of the radiologist) length and diameter: when a single vessel had both characteristics a single dominant vessel was identified (presence of a single dominant vessel); in some cases, two vessels had a comparable size and the radiologist decided for presence of two dominant vessels; in other cases, no vessel had “sufficient” size to be considered dominant (absence of dominant vessel). When a single dominant vessel was found the radiologist individuated its origin as “internal” or “lateral” thoracic; when two dominant vessels were found the origin was set to “internal and lateral” thoracic.

#### Semi-automatic evaluation

Image analysis included a blood vessel semi-automatic detection procedure. In order to obtain an optimal angiographic effect, the maximum intensity projection (MIP) was calculated by DCE-MRI data. A single vertical MIP projection was generated using the subtraction volume between the first post-contrast sequence and the pre-contrast sequence.

Semi-automatic evaluation of breast vascular map was previously reported in detail by Fusco et al. [16]. Briefly, our method is composed of two steps: first, a multi-scale vessel enhancement filtering, as reported in [17], was applied to the subtracted MIP; second, morphologic operators were applied on the resulting images in order to extract vessel skeletons.

**Fig 1:**
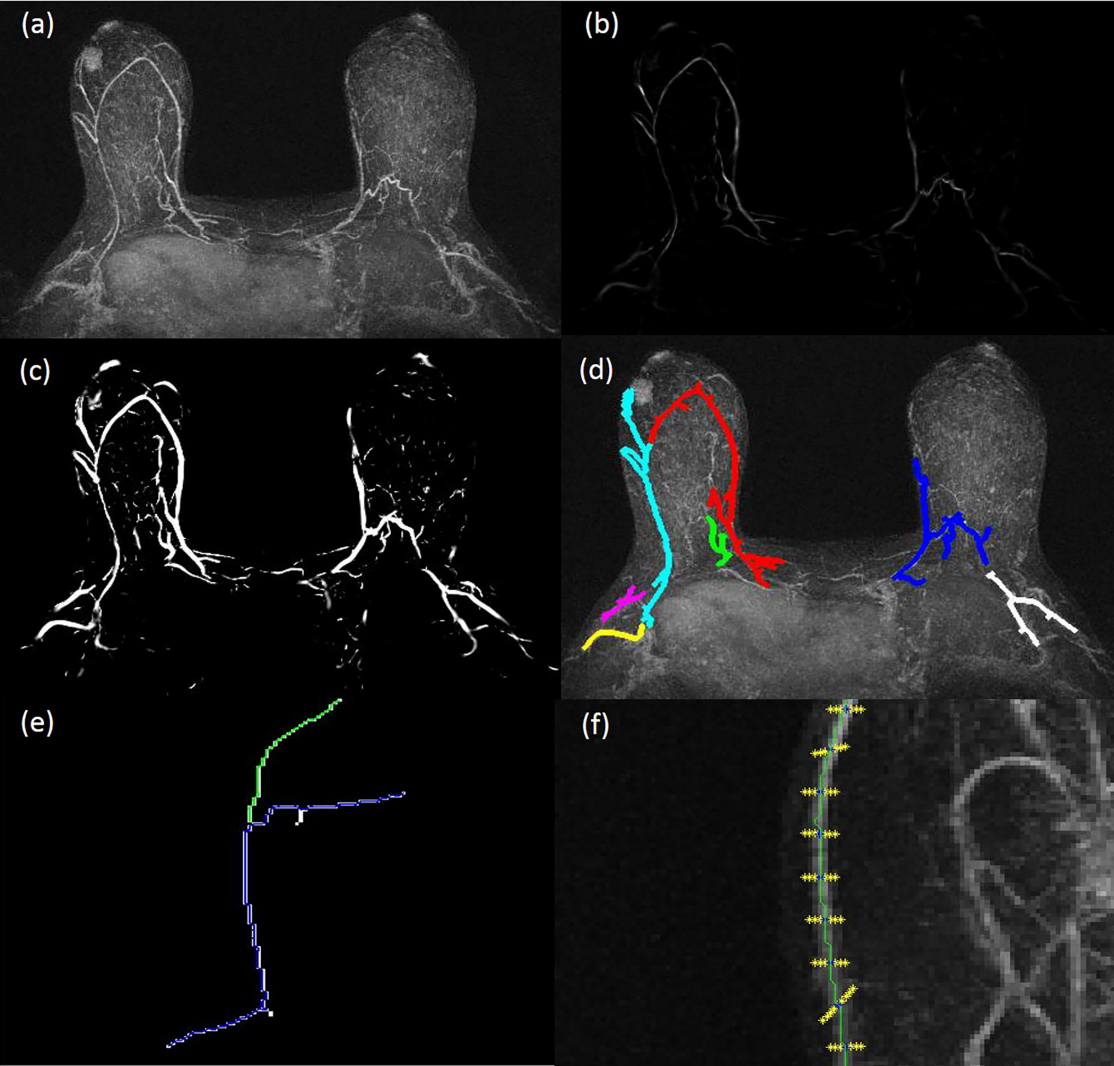
Semiautomatic vascular map extraction: (a) MIP obtained subtracting the first post-contrast sequence from pre-contrast sequence; (b) multiscale vessel enhancement filtering; (c) optimal contrast image after global histogram equalization; and (d) skeleton with extracted vessel in different colors (color figure online); (e) Semiautomatic vessel length and diameter measure: a vessel tracking; (f) normal segments to vessel direction.

In the first step, the local image structure of the subtracted MIP (Fig 1A) is analyzed at different scales using a “vesselness” measure involving local Hessian eigenvalues: per each pixel the “vesselness” can be considered the probability that it belongs to a vessel. In order to magnify the vascular map and to suppress background noise the “vesselness” image (Fig 1B) is processed by global histogram equalization (Fig 1C) and then converted into a binary (black-white) image using the Otsu’s global threshold method [18].

Morphologic operators were applied on the resulting image in order to detect connected regions (vessels) and to eliminate spurious pixels. Specifically, thinning operator was applied repeatedly until convergence: the obtained skeleton identified the longitudinal direction of vessels.

Length of vessels was measured along the longitudinal direction; vessel diameters were measured orthogonally to longitudinal direction.

In order to mimic radiologists criteria for dominant vessel identification, significant vessels were defined as having a length of at least 3 cm and a diameter of at least 2 mm [6–8, 19]. When no vessel had these requirements, no dominant vessel was identified. The dominant feeding vessel was identified as having the highest length among significant vessels. Dominant vessel was marked in red (Fig 1D).

Looking at this last image the radiologist identified the origin of vascular supply (internal or lateral thoracic). However, putting vessels in descending ordered based on diameter size, it was found that in some cases the first two vessels have very similar diameter: in the case this difference was no more than 5%, they were considered both as dominant vessels and the origin was set to both internal and lateral thoracic.

No training was performed by expert radiologist for both radiological and semi-automatic evaluation.

Image analyses were carried out using the MATLAB R2007a software (The MathWorks Inc., Natick, MA).

### Statistical Analysis

In order to evaluate variability between the two readers and between the radiologic consensus interpretation and the semi-automatic detection, inter-reading concordance assessment was assessed by means of Cohen's kappa coefficient (k) for categorical items (0–0.20, poor agreement; 0.21–0.40, fair agreement; 0.41–0.60, moderate agreement; 0.61–0.80, good agreement; and 0.81–1.00, excellent agreement). Cohen's kappa coefficient was used as metric to validate the predominant feeding vessel origin obtained by semi-automatic approach compared with qualitative perspective. Conbach's alpha [20] was used as a (lower bound) estimate of the reliability (internal consistence) of semi-automatic breast vascular map assessment (α<0.5 unacceptable, 0.5≤ α< 0.6 poor, 0.6≤ α< 0.7 acceptable, 0.7≤ α< 0.9 good, α≥ 0.9 excellent). Chi-square and Fisher exact test was used to assess statistically significant differences in the number of internal thoracic and lateral thoracic artery as predominant feeding vessel and correlation with lesion location. Median number of automatically detected vessels per each breast in malignant and benign lesions were compared using the Mann Whitney test for unpaired data. Chi-square test was used to assess a statistical significance between vascular map detection in the breast with lesion compared with the contralateral.

## Results

### Histological results

148 patients had malignant tumors (66.4%): 99 cases of invasive ductal carcinoma, 9 cases of invasive lobular carcinoma, 11 cases of mixed invasive ductal and invasive lobular carcinomas and 29 ductal carcinoma in situ. 75 patients had benign tumors (34.6%): 47 cases of fibroadenoma, 18 of atypical hyperplasia, six of fibrocystic change and four of intraductal papilloma (Table 1). In Table 2 were reported histopathological features of malignant lesions.

**Table 1:** Pathologic diagnosis of all tumors.

Malignant	N. Pts	Benign	N. Pts
IDC	99	Fibroadenoma	47
ILC	9	Fibrocystic changes	6
IDLC	11	Atypical Hyperplasia	18
DCIS	29	Intraductal Papilloma	4

*Note—*IDC = Invasive Ductal Carcinoma; ILC = Invasive Lobular Carcinoma; IDLC = Invasive Ductal-Lobular Carcinoma; DCIS = Ductal Carcinoma In Situ.

**Table 2:** Histopatologic features of malignant lesions.

Histopathologic Features	N. Pts.
ER/PR positive	52
HER2 positive	21
Triple positive	12
Triple negative	63

*Note—*ER = Estrogen Receptors; PR = Progesterone Receptors; HER2 = Human Epidermal Growth Factor Receptor 2.

### Lesion Location and presence of dominant feeding vessel

Table 3 shows lesion location and presence of dominant feeding vessel for malignant and benign lesions. Anatomic analysis of breast lesions revealed that 20% (44/223) of patients had masses in internal half, 50% (111/223) of patients had masses in external half and the 30% (68/223) of patients had masses in subareolar/central area.

**Table 3:** Tumor location and malignancy. Table entries: number of lesions.

		Malignancy	
		Malignant	Benign
**Tumor location**	Internal half	32	12
	External half	64	47
	Subareolar/Central Area	52	16

According to semi-automatic approach, the median number of vessels in the breasts containing malignant lesions was 3.9±1.6, compared with 2.8±1.4 in breasts containing benign lesions (p<0.05), and median length of predominant feeding vessel was 12.5±2.4 mm in patients with malignant tumors and 7.4±3.5 mm in patients with benign tumors. These differences were statistically significant (p<0.05 at Mann Whitney test). There were not differences statistically significant in the median number of vessels and in the median size (length and diameter) of predominant feeding vessel among different subgroups of malignant lesions (p>0.05 at Mann Whitney test). Instead there was a difference statistically significant (p<0.05 at Mann Whitney test) between fibroadenoma lesions and other benign lesions in the median number of breast vessel (2.9±1.5 versus 2.0±1.1) and in the median size of predominant feeding vessel (length 8.7±2.4 mm versus 6.3±1.8 mm; diameter 2.4±0.4 mm versus 2.1±0.3 mm) (Table 4). A dominant feeding vessel was present in 91% of malignant cases for both semi-automatic and radiologic evaluation, while predominant feeding vessel was present with lower percentage in benign lesion, respectively 67% and 65% (Table 4).

**Table 4:** Relationship between lesion subtype and size with dominant feeding vessels. The number of feeding vessels, the dominant vessel length and diameter were evaluated exclusively with the semi-automatic approach.

	Lesion type	Lesion Size [cm] (median±SD)	Number of feeding vessels (median±SD)	Dominant vessel length [mm] (median±SD)	Dominant vessel diameter [mm] (median±SD)	Presence of dominant vessel (semiautomatic)	Presence of dominant vessel (consensus reading)
Malignant	IDC	3.5±1.4	3.8±1.3	12.3±4.3	3.4±0.6	128	127
	ILC						
	IDLC						
	DCIS	1.3±3.8	3.1±1.1	11.5±4.9	3.2±0.5	7	7
Benign	Fibroadenoma	3.2±1.8	2.9±1.4	8.7±2.4	2.4±0.4	40	38
	Fibrocystic changes	2.4±1.1	2.0±1.1	6.3±1.8	2.1±0.3	10	11
	Atypical hyperplasia						
	Intraductal papilloma						

A good concordance between two radiologic assessments (k = 0.78 with 95% interval confidence 0.68–0.88) and an excellent agreement between radiologic consensus interpretation and semi-automatic assessment (k = 0.88 with 95% interval confidence 0.79–0.96) were reported to identify presence or absence of predominant feeding vessel.

### Dominant feeding vessel location and correlation with tumor location

As regards the 44 tumors in internal half, based on radiologic consensus interpretation, 40 demonstrated a predominant feeding vessel (61% were supplied by internal thoracic vessels, 14% by lateral thoracic vessels, 16% by both thoracic vessels and 9% had no predominant feeding vessel—p<0.01), based on semi-automatic detection, 38 tumors demonstrated a predominant feeding vessel (66% were supplied by internal thoracic vessels, 11% by lateral thoracic vessels, 9% by both thoracic vessels and 14% had no predominant feeding vessel—p<0.01). As regards the 111 tumors in external half, based on radiologic consensus, 91 demonstrated a predominant feeding vessel (25% were supplied by internal thoracic vessels, 39% by lateral thoracic vessels, 18% by both thoracic vessels and 18% had no predominant feeding vessel—p<0.01), based on semi-automatic detection, 94 demonstrated a predominant feeding vessel (27% were supplied by internal thoracic vessels, 45% by lateral thoracic vessels, 4% by both thoracic vessels and 24% had no predominant feeding vessel—p<0.01). The difference in the proportion of internal and lateral thoracic dominant vessels was statistically significant (p<0.01), for both internal and external masses, as shown in Table 5. No significant statistical difference was found between the proportion of internal and lateral thoracic artery when the masses were localized in subareolar/central area (Table 5).

**Table 5:** Tumor location and dominant feeding vessel origin findings for single reader, for consensus reading and for semi-automatic evaluation. Table entries: number of lesions.

		Dominant feeding vessel origin			
		Internal Thoracic	Lateral Thoracic	Internal and Lateral Thoracic	No predominant feeding vessel
		*Reader 1*			
**Tumor location**	Internal half	24	6	6	8
	External half	27	42	20	22
	Subareolar/Central Area	20	14	18	16
		*Reader 2*			
**Tumor location**	Internal half	25	5	7	7
	External half	27	42	19	23
	Subareolar/Central Area	19	16	17	16
		*Consensus reading*			
**Tumor location**	Internal half	27	6	7	4
	External half	28	43	20	20
	Subareolar/Central Area	20	15	17	16
		*Semi-automatic evaluation*			
**Tumor location**	Internal half	29	5	4	6
	External half	30	50	14	17
	Subareolar/Central Area	21	15	17	15

An excellent concordance between two radiologic assessments (k = 0.82 with 95% interval confidence 0.73–0.89) and an excellent agreement between radiologic consensus interpretation and semi-automatic assessment (k = 0.80 with 95% interval confidence 0.72–0.87) were reported to identify origin of predominant feeding vessel.

An excellent reliability for semi-automatic assessment (Cronbach's alpha equal to 0.96) was also assessed.

**Fig 2:**
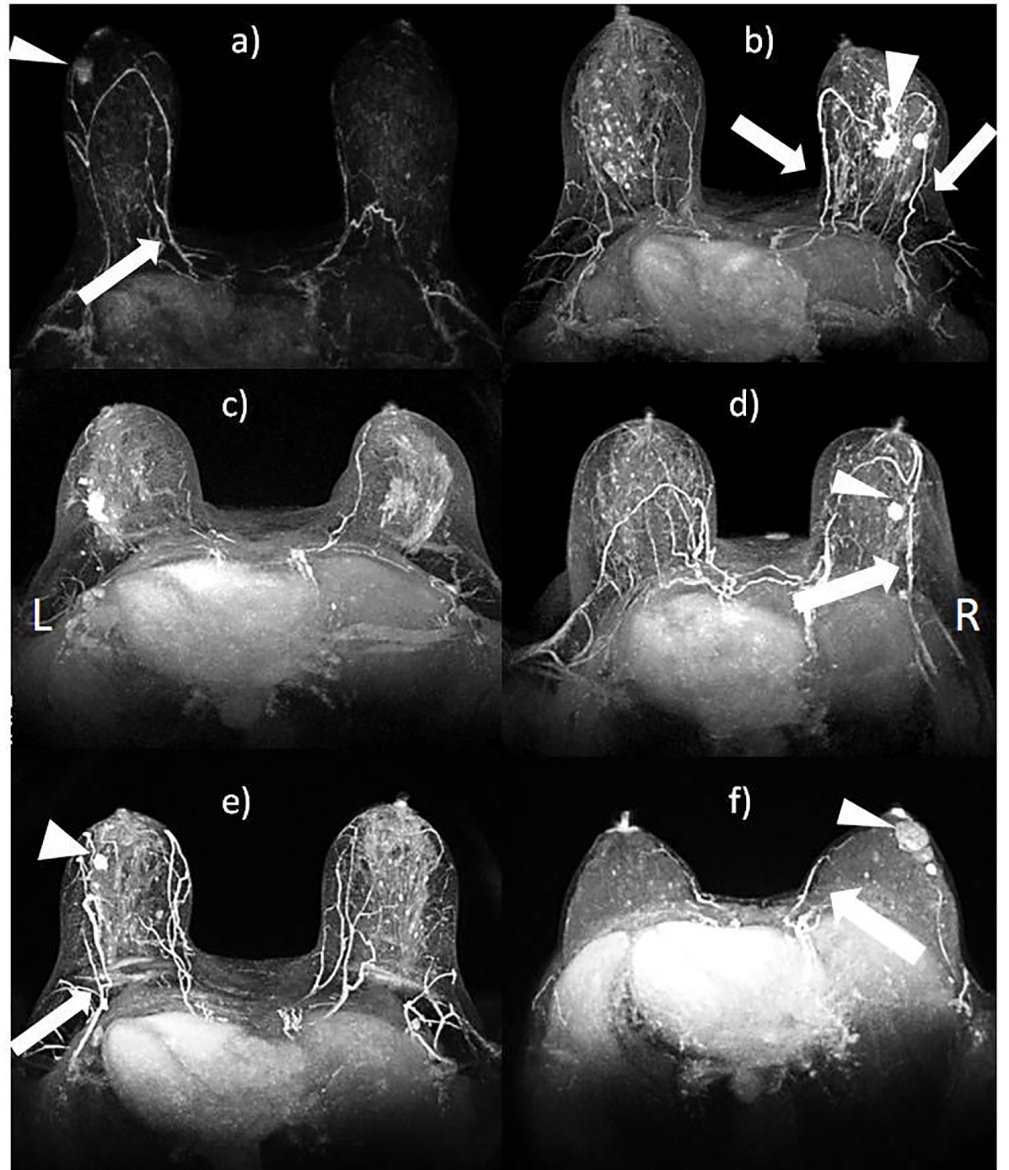
MIP reconstructions and predominant vessel: a) A lateral tumor (external quadrants) and dominant feeding vessel with origin in internal thoracic artery (automatic and radiologic assessment); b) A subareolar/central and lateral mass and dominant vessel with origin in internal and lateral thoracic artery (automatic and radiologic assessment); c) A lateral tumor (external quadrants) without dominant feeding vessel (automatic and radiologic assessment); d) A lateral tumor (external quadrants) with dominant feeding vessel with origin in lateral thoracic artery (automatic and radiologic assessment); e) A lateral tumor (external quadrants) with dominant feeding vessel with origin in lateral thoracic artery (automatic and radiologic assessment); f) A subareolar mass and dominant vessel with origin in internal thoracic artery (recognized only by automatic evaluation). Note- the triangle indicates the lesion and the arrow indicates the predominant feeding vessel.

Fig 2 shows examples of MIP reconstruction with tumor location and relative dominant vessel.

## Discussion

Angiogenesis is an important characteristic of breast carcinoma growth and it allows to observe differences in contrast enhancement between breast tumors and fibro-glandular tissue on CE-MRI [1–13]. The branches of internal thoracic artery provide most of the blood supply to the normal breast, especially to the inner compartments. The branches of lateral thoracic artery supply mainly the outer quadrants of the breast, as reported in [13].

MR is the best imaging modality for breast cancer detection and diagnosis. The location of the primary tumor influences survival in breast cancer, with a low outcome for tumors in inner and periareolar quadrants [21]. Specifically, patients with inner quadrant tumors had higher hazards for systemic disease relapse when compared to those located in the outer quadrants [21, 22]. We assumed that a breast with a malignant mass might show an altered blood supply according to the tumor location; this could be used as indicator of prognosis and survival and could be detected on the MIP obtained by MRI processing. In literature [1–13], the critical issues were the subjectivity of visual methods for vessel analysis and intra- and inter-observer variability of the predominant feeding vessel quantification. Orgüç et al [23] compared retrospectively bilateral 3D vascular maps obtained by dynamic CE-MRI and determined the association of one-sided vascular increase with ipsilateral breast cancer. They reported that MRI vascular mapping is an accurate method (sensitivity of 69% and specificity of 92%) for characterizing breast tissue vascularization. Radiological interpretation of predominant feeding vessel could be time consuming considering the need to measure the size of each vessel and then to identify the dominant vessel. In this scenario, it is desirable to assess the reliability of a semi-automatic approach for breast vascular map detection and for identification of predominant feeding vessel origin in correlation with tumor site.

Vignati et al [24–25] proposed an automatic algorithm for vessel detection from breast DCE-MRI and evaluated the clinical impact of this algorithm to assess neo-adjuvant therapy response. Their preliminary findings suggested the use of automatic vascular maps as biomarker to assess neo-adjuvant therapy pathologic response. Lin et al [26] developed a computer-based algorithm for detecting blood vessels appearing in breast dynamic CE-MRI analyzing 34 cases: the median correct-detection rate was 85.6% (mean 84.9% +/- 7.8%), the incorrect-detection rate was 13.1% (mean 15.1% +/- 7.8%), and the missed-detection rate was 19.2% (mean 21.3% +/- 12.8%). Both Vignati et al. [24–25] and Lin et al. [26] have not assessed the correlation between predominant feeding vessel and lesion location as a sign of malignity. In this study, we proposed a semi-automatic method to obtain breast vascular map and predominant feeding vessel and to correlate dominant vessel with lesion location. We have compared the findings of semi-automatic approach with the results of two readers expert radiologists evaluating the inter-observer variability.

The results indicated an excellent agreement between two radiologic assessments, radiologic consensus interpretation and semi-automatic assessment (k = 0.82 and k = 0.80, respectively); moreover, the findings showed that semi-automatic breast vascular map and predominant feeding vessel detection identified correctly, with excellent reliability, the lesion dominant vessel and its anatomic location (Cronbach's alpha equal to 0.96). Our investigation, based on semi-automatic assessment, revealed the presence of a prominent feeding vessel in 91% of the patients with a malignant tumor compared to 67% of patients with benign lesions (p<0.05 at Chi square test). Therefore, this latter could be used as sign of malignity. Moreover, our findings of semi-automatic approach revealed that an internal thoracic artery supplied a high percentage of the breasts with a tumor in internal half and that the lateral thoracic artery supplied a high percentage of the breasts with a lateral tumor (external half). These results were in accordance with Grubstein et al [13], reporting a predominant medial vascular supply connected to the internal mammary vessels in 87% of medial tumors and 48% of lateral tumors.

Some limitations of this study could be mentioned. Radiologists evaluated only subjectively the size of dominant vessels, therefore it was not possible to assess inter-observer variabilities regarding vessel size. The correlation of others factors on breast tissue vascularity such as age, menopausal situation, in which the measurements have been made, habit of smoking was not evaluated and could be investigated in next studies. Moreover, the tool was tested for specific situations including unilateral and unifocal histo-pathologically confirmed enhancing breast lesions. However, next endpoint is to assess the tool for bilateral and/or multifocal breast cancer. In these case using combination of different MIP projection such as anterior-posterior and right-left some limitations including overlapping lesions and predominant vessel could be resolved. Another limit is due to vessel size being measured on projection images (MIPs). This might potentially give distorted measures, in fact, for example, measuring the diameter of a vessel having elliptical section it can be projected on the minor axis giving a wrong estimation of the vessel diameter; similarly, when measuring vessel lengths we are neglecting curvature of the vessels which might be longer in reality. Our analysis on MIP images implicitly assumed that vessel had circular section and curvature could be neglected. Future algorithm developments should consider a full 3D processing. The study is a retrospective study and we are not able to increase sample size; the dataset could be considered “unbalanced” (high number of low-vascular tumours is less than the number of high-vascular tumours) in the sense of pattern recognition jargon. It is well known that the performance of an algorithm tested on an “unbalanced” dataset could be misleading and the algorithm might be poorly generalizable. However, we considered retrospectively a study population of more than 3000 subjects from which we excluded a large number of patients in order to extract a homogeneous sample. From a statistical point of view, the sample size of this study (223 patients) allowed to perform all the statistical tests reported in the manuscript at a level of significance p<0.05 or better.

Clinical impact of this study could be assessed observing in a prospective study the added diagnostic value of local and global breast vascularity in comparison with standard MR functional descriptors (such as wash-in and wash-out rate) on large consecutive series of patients for prognosis, early and presurgical therapy response assessment in advanced breast cancer. Moreover, it might interesting to evaluate the vascularity of breast tissue using MRI angiography in correlation with Doppler Ultrasound. Finally, it must be highlighted that, in this preliminary study, we focussed on defined and clearly visible breast lesions in order to evaluate the feasibility of the semi-automatic approach; in future prospective studies hard to evaluate studies will be included in order to assess the clinical impact of the method.

## Conclusions

This study demonstrated that semi-automatic breast vascular map and predominant feeding vessel detection can identify correctly with a good reliability the lesion dominant vessel and its anatomic origin. Results of semi-automatic vascular map assessment showed a differential blood supply to breasts with malignancies according to the tumor location and an increased vascularity compared to breasts without malignant tumors: internal thoracic artery supplied the highest proportion of breasts with a tumor in internal half and lateral thoracic artery supplied the highest proportion of breasts with a lateral tumor.
